# Use of Submicron Vaterite Particles Serves as an Effective Delivery Vehicle to the Respiratory Portion of the Lung

**DOI:** 10.3389/fphar.2018.00559

**Published:** 2018-06-04

**Authors:** Olga Gusliakova, Elena N. Atochina-Vasserman, Olga Sindeeva, Sergey Sindeev, Sergey Pinyaev, Nikolay Pyataev, Viktor Revin, Gleb B. Sukhorukov, Dmitry Gorin, Andrew J. Gow

**Affiliations:** ^1^Remote Controlled Theranostic Systems Lab, Saratov State University, Saratov, Russia; ^2^RASA Center in Tomsk, Tomsk Polytechnic University, Tomsk, Russia; ^3^RASA Center, Kazan Federal University, Kazan, Russia; ^4^Department of Biotechnology, Bioengineering and Biochemistry, National Research Ogarev Mordovia State University, Saransk, Russia; ^5^School of Engineering and Materials Science, Queen Mary University of London, London, United Kingdom; ^6^Skoltech Center for Photonics and Quantum Materials, Skolkovo Institute of Science and Technology, Skolkovo Innovation Center, Moscow, Russia; ^7^Pharmacology and Toxicology, Rutgers University, Piscataway, NJ, United States

**Keywords:** vaterite particles, pulmonary drug delivery, prolonging release, size-dependent biodistribution, drug carriers

## Abstract

Nano- and microencapsulation has proven to be a useful technique for the construction of drug delivery vehicles for use in vascular medicine. However, the possibility of using these techniques within the lung as an inhalation delivery mechanism has not been previously considered. A critical element of particle delivery to the lung is the degree of penetrance that can be achieved with respect to the airway tree. In this study we examined the effectiveness of near infrared (NIR) dye (Cy7) labeled calcium carbonate (vaterite) particles of 3.15, 1.35, and 0.65 μm diameter in reaching the respiratory portion of the lung. First of all, it was shown that, interaction vaterite particles and the components of the pulmonary surfactant occurs a very strong retardation of the recrystallization and dissolution of the particles, which can subsequently be used to create systems with a prolonging release of bioactive substances after the particles penetrate the distal sections of the lungs. Submicro- and microparticles, coated with Cy7 labeled albumin as a model compound, were delivered to mouse lungs via tracheostomy with subsequent imaging performed 24, 48, and 72 h after delivery by *in vivo* fluorescence. 20 min post administration particles of all three sizes were visible in the lung, with the deepest penetrance observed with 0.65 μm particles. *In vivo* biodistribution was confirmed by fluorescence tomography imaging of excised organs post 72 h. Laser scanning confocal microscopy shows 0.65 μm particles reaching the alveolar space. The delivery of fluorophore to the blood was assessed using Cy7 labeled 0.65 μm particles. Cy7 labeled 0.65 μm particles efficiently delivered fluorescent material to the blood with a peak 3 h after particle administration. The pharmacokinetics of NIR fluorescence dye will be shown. These studies establish that by using 0.65 μm particles loaded with Cy7 we can efficiently access the respiratory portion of the lung, which represents a potentially efficient delivery mechanism for both the lung and the vasculature.

## Introduction

The delivery of drugs to the lower respiratory portion of the lung is a long standing and desirable goal. The distal portion of the lung is a desirable target for systemic delivery as it has a large surface area (∼70–140 m^2^); the narrow barrier for diffusion, and relative lack of degradative enzymes (Groneberg et al., 2003). In addition, the delivery of compounds to the respiratory portion is an important goal for many pulmonary diseases. According to the Global Burden of Disease Study project, four of the fifteen most prevalent causes of death occur in the respiratory portion of the lung. In the forecast for 2030, all these diseases are anticipated to increase in relevance (Mathers and Loncar, 2006). For the treatment of these diseases, the design of delivery systems aimed specifically at the distal lungs is potentially of great value.

The distribution of inhaled particles within the lungs is dependent upon a number of physical forces (such as inertia, gravity, and Brownian motion) and biophysical interactions (such as interaction with the lung lining fluid and cells of the epithelial surface). Particle size is a critical determinant in the response to many of these factors. Particles of large size >5 μm are more susceptible to inertial forces, and hence these particles distribute predominantly in the upper conducting airways. For the distribution of particles of smaller size gravitational and Brownian forces become more relevant. Leading to 1–5 μm particles being distributed at the bronchiolar level, and smaller particles (<1 μm) reaching the distal alveolar portion. However, smaller particles are greater risk of being exhaled and may have overly rapid dissolution characteristics, depending on their interaction with the lung lining (Patton and Byron, 2007; El-Sherbiny et al., 2015). To avoid these drawbacks, it is the goal of this study to find the largest particle size that can be efficiently delivered to the distal portion of the lung. To date, a number of particles have been considered for pulmonary delivery (Lowry et al., 2007; Smola et al., 2008; Nokhodchi and Martin, 2015), but for this study, we examined vaterite particles as they have a potentially advantageous interaction with the lung lining.

It is relatively simple to generate vaterite particles in a range of sizes (300 nm to 6 μm) and they are generally biocompatible (Biradar et al., 2011), with low toxicity (Parakhonskiy et al., 2015; Svenskaya et al., 2016b), a high loading volume, and dissolute in relatively mild conditions (pH < 5.5) (Ma and Zhu, 2010; Svenskaya et al., 2013). All of these characteristics make them attractive as a delivery system for the treatment of either local lung diseases or for slow delivery to the systemic circulation. Vaterite particles are considered to hold promise in the drug delivery field as a controlled release mechanism (Trushina et al., 2014; Volodkin, 2014; Svenskaya et al., 2016b). Indeed, calcium carbonate microparticles have been used for both nasal (Borodina et al., 2016) and transdermal delivery (Genina et al., 2016; Svenskaya, 2017). In the case of transdermal delivery, it should be noted that the use of microneedles may greatly improve efficiency (Ye et al., 2016, 2017). Recently, microparticles formed from inorganic salts (calcium carbonate and calcium formate) and incorporating hyaluronan were examined as a potential aerosol delivery mechanism with a resulting increase in bioavailability (Tewes et al., 2016).

Vaterite particles can be loaded with a variety of active substances, which leads to distribution both on the surface and inside the particles due to their porous structure. Furthermore, such particles can be coated with polyelectrolytes to regulate their interaction with biological materials. Synthesized vaterite particles are mostly polycrystals that have spherical or ellipsoidal shape with a large number of pores ranging in size from 30 to 50 nm (Sukhorukov et al., 2004). The most stable form of vaterite is calcite crystals and thus dissolution of these particles leads to recrystallization. This process of recrystallization is accompanied by the complete release of substances previously contained in the particles (Parakhonskiy et al., 2012; Parakhonskiy B. et al., 2013; Parakhonskiy B.V. et al., 2013). It has been shown that vaterite particles are capable of interacting with proteins in their environment to form a corona, which preserves the particle in its initial state, slowing down the process of transformation into calcite (Kreyling et al., 2015; Sergeeva et al., 2015; Soliman et al., 2015). The lung lining fluid consists predominantly of phosphatidylcholine but does contain four specific surfactant proteins. It is our hypothesis that administration of vaterite particles will lead to the generation of a corona made up of these biomaterials that will reduce recrystallization and slow the release of particle contents. In this study, we have examined how altering the size of vaterite particles alters their distribution within the lung. Further we have measured how these changes alter the pharmacokinetics of delivery of these materials both to the lung and the body as whole. In the long term the goal of this research is to design vaterite particles that can be used for effective drug delivery with good biocompatibility, low toxicity, and favorable pharmacokinetics.

## Materials and Methods

### Materials

Calcium chloride (CaCl_2_), sodium carbonate (Na_2_CO_3_), sodium chloride (NaCl), ethylene glycol, phosphate buffered saline (PBS), bovine serum albumin (BSA) were purchased from Sigma-Aldrich (St. Louis, MO, United States) and Cyanine 7 NHS ester (Cy7) was purchased from Lumiprobe (Moscow, Russia). All chemical agents were used without further purification. Deionized water produced with a water treatment system Milli-Q (Millipore, United States) was used in all experimental stages.

### Synthesis of Vaterite Particles

It is sufficient to mix two salts (calcium chloride and sodium carbonate) in equal amounts to obtain vaterite particles. By changing the synthesis conditions (the method of mixing, the presence of additional agents, the temperature, etc.), it is possible to control the size of the produced particles.

Three methods were used to prepare particles of three sizes: 3.15, 1.35, and 0.65 μm. Spherical microparticles of calcium carbonate 3.15 μm in diameter were prepared as previously described (Volodkin et al., 2004b). Briefly, solutions of sodium carbonate (0.33 M) was continuously mixed with calcium chloride (0.33 M) in an equal volume for 1 min at a rate of 500 rpm on magnetic stirrer. Vaterite particles with the size of 0.65 μm were prepared according to the protocol as previously described (Parakhonskiy et al., 2012). Briefly, ethylene glycol was added to the reaction mixture as described above and solution was mixed for 3 h at 650 rpm on magnetic stirrer. Adding of ethylene glycol diminished the molecular diffusion, reducing the crystal growth rate and the probability of nucleation, which finally stabilized the vaterite crystals and eventually leads to a decrease in the particles size. Vaterite particles with the size of 1.35 μm were prepared in combination of two methods: increasing the viscosity of the reaction mixture by adding ethylene glycol, as described in the previous method, and using ultrasonic mixing, as previously described (Svenskaya et al., 2016a) for synthesis in water. The mixture of two salts and ethylene glycol as solvent in the ratio of 17 and 83%, respectively, was stirred for 15 min under the sonication by the ultrasonic homogenizer Sonopuls (Bandelin, Germany) at the frequency of 20 kHz and power density of 1 W/cm^2^. Due to the continuous operation of the ultrasonic homogenizer, the temperature of the reaction mixture increased significantly. To reduce this effect, the glass with the mixture was placed in cold water. The water temperature was maintained at 10°C by the adding ice. Thus, a temperature gradient was obtained from 33°C around the ultrasonic homogenizer rod to 10°C near the glass walls. In this case, a local increase in temperature led to an increase in the vaterite particle size by a factor of 2 compared to the methods where stirring of the reaction mixture in the presence of ethylene glycol was carried out on a magnetic stirrer.

After each synthesis, particles were precipitated by centrifugation for 1 min at 3,000 *g* for micron particles and at 6,000 *g* for submicron particles and subsequent thrice washed with deionized water and a single wash with ethanol. The resulting precipitate was then dried for 1 h at +60°C. To study the morphology and microstructure, dried particles were sputtered with gold and imaged with scanning electron microscopes (SEM), a MIRA II LMU (Tescan) at an operating voltage of 30 kV.

### Labeling Vaterite Particles With Conjugated Cy7-BSA

Fluorescent dye of Cy7 was dissolved in anhydrous DMSO (4:1), added to 50 ml of 2% BSA in PBS, pH 8.3 and stirred overnight at +4°Ñ. The resulting Cy7 conjugated BSA solution was washed from excess reagents by extensive dialysis in water. The conjugated Cy7-BSA (2 ml) was mixed with 5 mg of the obtained dried CaCO_3_ particles and incubated during 1 h of shaking at room temperature. Micron-sized particles were centrifuged at 3,000 *g* for 1 min, sub-micron particles were centrifuged at 6,000 *g* for 1 min. Afterwards the supernatants were removed and collected. The concentration of the fluorescent dye was determined photometrically using a spectrophotometer (Synergy H1) according to the calibration line determining the relative fluorescent units (RFU) dependence and the known concentration. A confocal microscope Leica TCS SP8 X (Leica Microsystems) was used to visualize the obtained labeled vaterite particles.

### Interaction of Vaterite Particles With Surfactant *in Vitro*

The obtained vaterite particles at a size of 0.65 μm were incubated at the concentration 10 mg/ml with deionized water, saline, small or large aggregate surfactant fractions with constant shaking at 37°C at indicated time points (1, 3, 5, 7, 9, 24, 32, 56, 96, and 144 h), the samples were analyzed for the particle morphology with scanning electron microscopy MIRA II LMU (Tescan).

### Animals

All animals for this study were housed in the Animal Care Facility of Ogarev Mordovia State University under standard conditions with free access to food and water. All animal procedures were performed according to a protocol approved by the Institutional Animal Care and Use Committees of the Medical Institute of Ogarev Mordovia State University (Ethics Committee protocol # 50 from 20.05.2017). Balbc mice, mix population of 6–8 week-old, were euthanized with Zoletil mixture (40 mg kg-1, 50 μl, Virbac SA, Carros, France) and 2% Rometar (10 μl and 10 mg kg-1, Spofa, Czechia) via intraperitoneal injection.

### Preparation of Bronchoalveolar Lavage

Bronchoalveolar lavage (BAL) was performed with 0.5-ml aliquots of sterile saline to a total volume of 10 ml as described (Guo et al., 2008). Recovered BAL samples were centrifuged at 400 *g* at 4°C for 10 min to remove cells, and cell-free BAL supernatants were separated into large- (LA) and small- (SA) aggregate surfactant fractions by centrifugation (20,000 *g* for 60 min at 4°C) as described previously (Atochina et al., 2004). Pelleted a biophysically active LA fraction was resuspended in sterile saline and frozen at -20°C until analysis. The supernatant from the high-speed centrifugation, SA fraction that contained soluble proteins and biophysically inactive surfactant forms transferred to a fresh tube and also frozen at -20°C for future analysis.

### Biodistribution of the Vaterite Particles *in Vivo*

BSA-Cy7 labeled particles (0.65, 1.35, and 3.15 μm) were administered intratracheally to 6–8 weeks old Balb/c mice as described previously (Atochina-Vasserman et al., 2009). BSA-Cy7 conjugate alone was used as control, the dose of fluorescent dye Cy7 was 300 ng for each injection. All the mice (*n* = 3–4 per each group) were imaged prior injection and 5, 20 min and 24, 48, and 72 h post-injection. After intratracheal instillation behavior and well-being of mice remained normal and breathing was steady under anesthesia. After all the manipulations mice physical activity and appetite were normal for 3 days before they were sacrificed.

At indicated time after particles administration (24, 48, and 72 h), biodistribution of the fluorescent signal within lungs, liver, kidneys, stomach, and intestines of alive and anesthetized mice was analyzed using IVIS^®^ Lumina imaging system (Xenogen Corp.) with a ICG filter set (excitation, 710–760 nm; emission, 810–875 nm). All the fluorescence images were acquired with 5 s exposure and were normalized by dividing the fluorescent images with reference illumination images. Luminescence intensity was quantified using Living Image software (Xenogen Corp). To obtain numerical data on the level of intensity in the region of interest, post-processing of images was analyzed using the Fiji software^1^. The resulting level of fluorescence of the region of interest was calculated as the difference between the Radiant Efficiency at a certain time point and the initial level (time 0) for each mouse.

### Confocal Fluorescence Imaging of Lung

Twenty minutes post administration of 0.65 μm size particles/BSA-Cy7, mice were sacrificed, lungs were removed, washed with saline and frozen in a Leica cryostat with tissue freezing medium. The prepared 15 μm thickness sections was analyzed on a laser scanning confocal microscope (Leica TCS SP8 X). The laser was excited at 670 nm. The images were recorded using two fluorescent channels: 680–726 nm spectra range corresponding to autofluorescence of lung tissue and 747–794 nm spectra range, corresponding to the fluorescent dye Cy7. Optical images of the sample were also recorded.

*z*-stack technology was used for the local tissue 3-D visualization where the vaterite particles deposited in the alveolar space. After selecting the area of interest on the cryosection of the lung sample with instilled particles coated by fluorescent dye, a *z*-axis scanning range perpendicular to the cryosection plane was performed. The step of forming the confocal planes was not more than 0.2 μm. Following the completion of the sample scanning procedure in the specified range, the Las X Leica software carried out a 3-D reconstruction of the region of interest. The recording of fluorescent images was made in the same channels, which are described in a paragraph earlier.

### Pharmacokinetics

Mice were intratracheally injected with 0.65 μm size particles adsorbed with fluorescent dye Cy7 from the alcohol solution or with BSA-Cy7- conjugate. Fluorescent dye Cy7 in 0.01% alcohol solution was used as a control. The dose of the Cy7 dye in each case was 300 ng. At indicated time points (5, 15, and 45 min, 1.5, 3, 6, 9, 24, and 48 h) after administration, 50 μl of blood samples were taken through the retroorbital sine through a glass hematocrit capillary, mixed with heparin at a ratio of 5/3. The relative fluorescence units (RFU) intensity was measured by Synergy H1 spectrophotometer (BioTek Instruments, Inc.) with the excitation occurred at 720 nm, the emission spectrum at 750–800 nm with steps of 1 nm. The emission spectrum of the fluorescent dye Cy7 has a peak at 773 nm. For each concentration, it was calculated how much the value of the relative fluorescence unit (RFU) increased in comparison with the zero concentration in the indicated range, that is, the following ratio was calculated:

$$\begin{array}{c} I=\;\frac{\sum_{\lambda \;=\;765}^{785}RFU_{\lambda }^{\mathrm{dyed}}}{\sum_{\lambda =\;765}^{785}RFU_{\lambda }^{\mathrm{blank}}} \end{array}$$

Where “I” is a coefficient of intensity increase and “λ” - spectra wavelength. The summary of RFU signal was calculated from 765 to 785 nm in 1 nm steps.

### Statistics

For the ANOVA test, the site was used http://vassarstats.net (One-Way Analysis of Variance for Independent or Correlated Samples) which also perform pair-wise comparisons of sample means via the Tukey HSD test which compares all possible pairs of means and uses Studentized range distribution. Two pairs (water/saline and Small/Large aggregate fraction) were compared to determine the statistical significance of obtained results in studying of the recrystallization dynamics of the vaterite particles in four different solutions. In biodistribution study all Radiant Efficiency values for individual organs in each group were compared in each time point (24, 48, and 72 h). The curves of pharmacokinetics for three different substances administration were compared. Two levels of significance were established (*p* < 0.05 and < 0.01).

## Results

**FIGURE 1:**
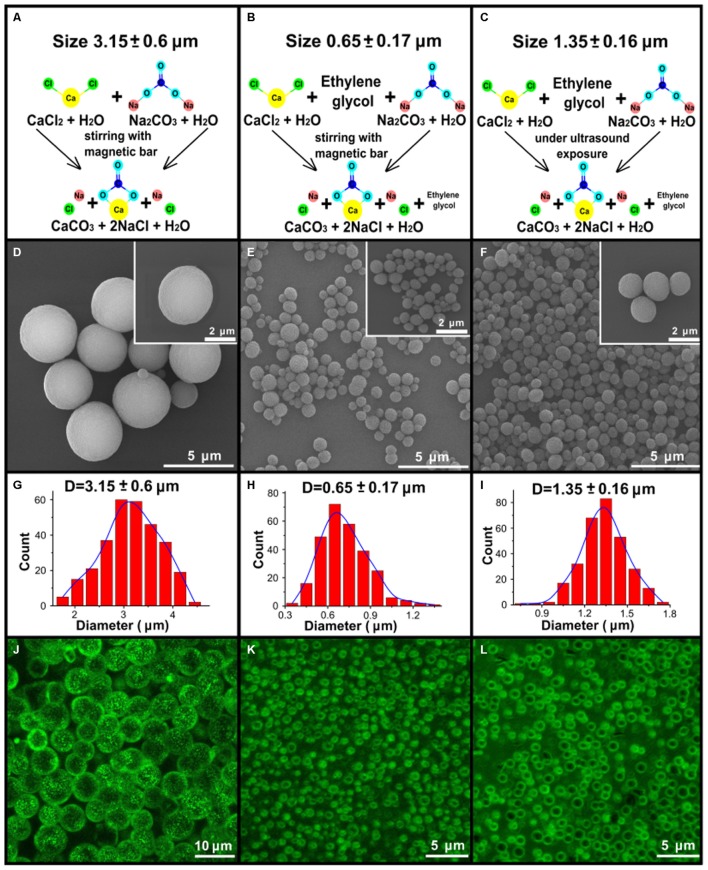
Synthesis and characterization of the vaterite particles. **(A–C)** Schematic presentation of the three types of synthesis to obtain 3.15, 0.65, and 1.35 μm vaterite particles as described in the “Materials and Methods” Section. **(D–F)** The scanning electron microscopy (SEM) images for vaterite particles with size diameters of 3.15, 0.65, and 1.35 μm vaterite particles. **(G–I)** The obtained particles size distributions. The diameters of 300 particles were measured using the free software ImageJ. **(J–L)** The fluorescent images of particles adsorbed with BSA-Cy7- conjugate, obtained by means of laser scanning confocal microscopy.

One of the goals of this study was to generate porous vaterite particles of varying diameter as we proposed that particle size would affect both distribution and dissolution within the lung. **Figure 1** shows the schemes (A–C) for the three types of synthesis used to obtain vaterite particles of different sizes. Segments (D–F) show images of particles obtained by scanning electron microscopy. Below each SEM image the distribution of particles size is shown (G–I). These distributions were obtained by the diameter of 300 particles within the SEM image with Fiji software. As one can see the three methods produce particles of three different size distributions averaging 3.15, 1.35, and 0.65 μm in diameter. The porous structure of the vaterite particles allows one to consider them as vehicles for soluble materials, such as biologically active substances. Loaded into the particles through the pores will allow these materials to be retained there when the particles are introduced into the body until the vaterite will be recrystallized or dissolved. To examine how well our particles retain materials we examined loading them with BSA conjugated to the fluorescent dye Cy7. **Figures 1J–L** show confocal images of loaded particles that demonstrate labeling with Cy7 and that the concentration of dye is greatest at the vaterite shell. **Supplementary Figure S1** demonstrates normalized fluorescence intensity spectrum and schematic of the Cy7 molecular structure. The estimation of the encapsulation efficacy of these vaterite containers showed that the highest loading was obtained on particles with the smallest size of 0.65 μm and it was 10.3% w/w compared to 1.5 and 0.88% w/w for particles with a size of 1.35 and 3.15 μm, respectively. It should be noted that in this study, absorption was performed in one cycle, since it was not our aim to obtain the maximum amount of the conjugate (Cy7-BSA) absorbed on vaterite particle. It is possible to improve this index by carrying out several absorption cycles. Studies of fluorescent dye biodistribution and pharmacokinetics were performed at the same doses of Cy7 for all types of instilled substances, which were 300 ng. A different number of vaterite particles were introduced for each size due to the different absorption efficiencies. 2.9 μg of vaterite was injected into the lungs in case of the smallest particles injection which is an order of magnitude less than for other particle sizes (20 and 34 μg for particles with a size of 1.35 and 3.15 μm, respectively).

**FIGURE 2:**
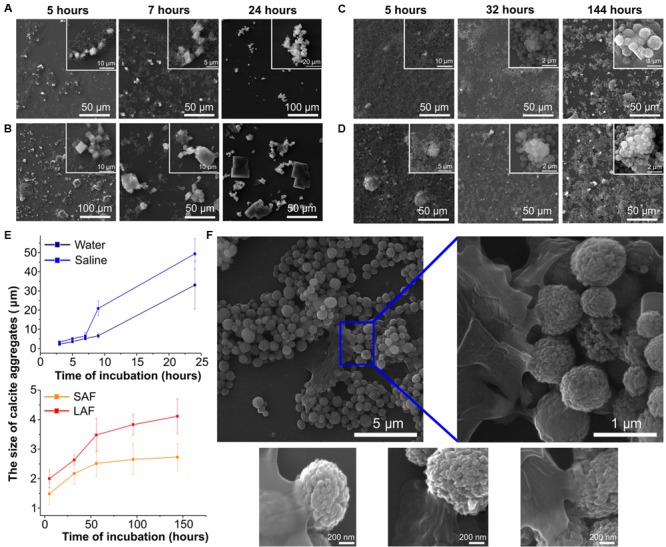
Interaction of vaterite particles with surfactant *in vitro*. SEM images of the particles incubated with **(A)** deionized water, **(B)** saline, **(C)** small, and **(D)** large aggregate surfactant fractions at indicated time. **(E)** Time-dependent changes of the size of calcite aggregates during incubation of the 0.65 μm vaterite particles with various biosolutions, significant difference (*p* < 0.01) appears after 7 h incubation in water and saline but for pair small (SAF) and large aggregate surfactant fraction (LAF) the significant difference (*p* < 0.05) takes place after 56 h of incubation. **(F)** SEM images of vaterite particles coated with LA surfactant fraction 56 h post incubation.

As mentioned previously, vaterite particles like any other foreign particles are capable of interacting with biological materials to form a corona (Nokhodchi and Martin, 2015). Therefore, we examined how incubation with the components of the lung lining fluid, the first point of contact for a pulmonary delivered compound, alters the morphology of the vaterite particles. Therefore, particles were incubated with water, saline solution, and the large (hydrophobic) and small (hydrophilic) aggregate surfactant fractions of the lung lining fluid and the rate of recrystallization assessed by SEM. Understanding how the components of the lung lining affect the rate of particle recrystallization is important in terms of the possibility of prolonging the release of biologically active substances. As can be seen from **Figure 2**, the morphological changes of the particle surface during incubation in deionized water and saline occur much faster than in the case of incubation with the lung lining fluids (A–E). Within 9 h of particle incubation with saline, and 24 h with water, calcium carbonate is present only in one form – calcite. In the construction of SEM images, the number of secondary electrons arriving at the detector (the result of the interaction of the electron beam and the investigated surface) is converted into a picture in gray tones. A different number of secondary electrons will be detected from surfaces with different chemical composition, although this dependence is not strong as for scattered electrons. However, a chemical element with a large atomic number on the pictures will have a lighter shade of gray (Goldstein et al., 2017). Thus, all cubic objects in **Figure 2B** having a dark gray color should be identified as salt crystals. Crystals of calcite, in turn, are distinguished by a bright light gray color. In the case of interaction with the lung lining components, the process of the vaterite transformation into calcite is significantly slowed down. One can see that even after 144 h of incubation, practically all particles are still present in the form of a vaterite. However, from the SEM images, it can be seen that the surface of some particles appears to have formed a corona when incubated with lung lining components (**Figure 2F**). From the **Figure 2E**, the size of the calcite particles in the case of incubation with the large aggregate surfactant fraction are two times larger than when incubating in the small aggregate surfactant fraction; suggesting that the hydrophilic components of the lung lining are more readily able to coat vaterite particles. Thus, it can be concluded that, considered interaction of the vaterite particles and the pulmonary surfactant component lead to prolonging release of the active substance that was previously loaded into the particles.

To obtain some quantitative evaluation of the recrystallization process, the graphs in **Figure 2E** were plotted. At each time point, for each sample, the geometric dimensions of calcite particles aggregates were measured using the free Fiji software, since for long times it is already impossible to visually determine the beginning and end of single calcite crystal due to their great compactness. By the first hour of incubation in deionized water and physiological solution there are still separate calcite crystals 3 μm in size and after 24 h their aggregate size exceeds 20 μm. While for incubation in fractions of pulmonary surfactant the growth of the sizes of both individual calcite particles and their aggregates occurs very smoothly, varying from 1.5 μm after 5 h to 2.7 μm after 144 h when particles interact with small aggregate surfactant fraction, in the case of vaterite incubation in a large aggregate surfactant fraction their growth varies from 2 μm to 4.1 over the same time period.

**FIGURE 3:**
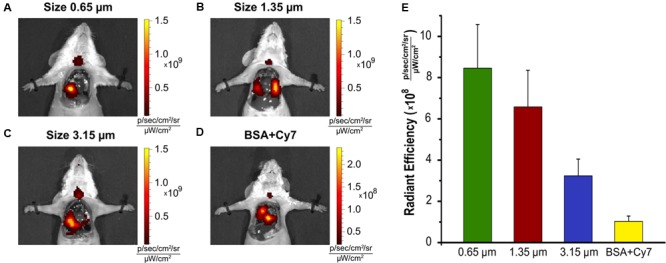
Biodistribution of the vaterite particles in the lung. Various size of the particles **(A)** 0.65 μm, **(B)** 1.35 μm, **(C)** 3.15 μm adsorbed with BSA-Cy7-conjugate, and **(D)** BSA-Cy7 alone were instilled through the tracheostomy. Mice were sacrificed 20 min post-instillation, the chest was open and a fluorescence imaging of the lung was performed for each animal. **(E)** The histogram of the average Radiant Efficiency within the target organ. The dose of fluorescent dye Cy7 was 300 ng for each injection. BSA-Cy7 conjugate alone was used as control. Data are expressed as mean value ± SD, *n* = 3 mice per group.

One of the factors affecting the distribution of particles in the lungs is their size, since different forces are responsible for the deposition of particles with different size. In this connection, three particle sizes were examined in this study. Particles of 0.65 μm size are expected to be distributed in the lungs owing to Brownian motion. Sedimentation will have a significant effect on particles of 1.35 μm size. The deposition of large particles (3.15 μm) should be most affected by the impact (El-Sherbiny et al., 2015). In addition, we considered the anatomy of the lung. The goal was to deliver particles to the distal portion and the size of the alveolar neck is approximately 1 μm. Therefore, we have not used particles considerably smaller than 0.5 μm as such particles would have no greater access to the distal portion but would be more susceptible to exhalation and rapid dissolution. Our proposal is that vaterite particle size will alter its distribution within the lung. Therefore, we examined the distribution of Cy7-labeled particles using *in vivo* fluorescent imaging (IVIS) following intratracheal instillation (**Figure 3**). Twenty minutes after instillation, all three particle sizes displayed significant distribution throughout the lung and to a greater extent than BSA-Cy7 alone. These results demonstrate that all the particles have the ability to retain the labeled material within the lung. However, the ability to retain appears to be size dependent as 0.65 μm particles show the greatest degree of retained fluorescence 20 min after instillation. This is born out over time where the smallest particles continue to retain dye within the lung (**Supplementary Figure S2**).

**FIGURE 4:**
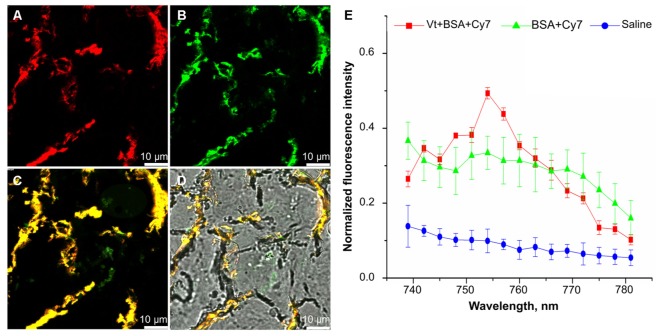
Confocal fluorescence images of lung cryosection. Lung sections were analyzed on a laser scanning confocal microscope 20 min post intratracheal administration of 0.65 μm particles adsorbed with BSA-Cy7 conjugate. Shown a representable lung section (*n* = 3–5 lung section per mouse, *n* = 3 mice). **(A)** a fluorescent signal in a range spectra of 680–726 nm, corresponded to autofluorescence of lung tissue. **(B)** A fluorescent signal in a range spectra of 747–794 nm, corresponded to fluorescent dye Cy7. **(C)** Combination of two channels from **(A,B)**. **(D)** Overlay of the optical image and two fluorescent channels as shown on **(C)**. **(E)** Emission spectra of lung tissue 20 min after intratracheal administration of 0.65 μm particles adsorbed with BSA-Cy7 conjugate (red curve), BSA- Cy7 alone (green curve) or saline (blue curve). Data are expressed as mean value ± SD, *n* = 5 spectra intensity point per each administrated substance.

**FIGURE 5:**
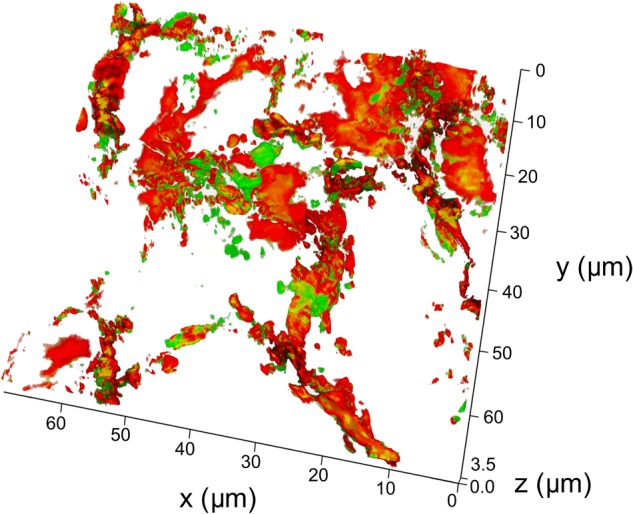
3D reconstruction of a lung cryosection fluorescent image. The particles of the 0.65 μm adsorbed with BSA-Cy7-conjugate were intratracheally administrated and mice were sacrificed 20 min post-instillation. Scanned confocal images of lung sections (*n* = 3–5 lung sections per mouse, *n* = 3 mice) were 3D reconstructed and analyzed. Thickness of 3D reconstruction was 3.5 μm. The step of constructing confocal planes was 0.2 μm. Shown representative image of 3D reconstructed lung section.

In order to determine whether particles did reach the respiratory portion of the lung, we examined the lung for presence of Cy7 using confocal fluorescent imaging (**Figures 4, 5**). Lungs were prepared for fluorescent imaging 20 min after intratracheal instillation of 0.65 μm vaterite particles loaded with BSA-Cy7 conjugate. Overlaying the signal obtained in the Cy7 channel (**Figure 4B**) over the tissue autofluorescence (**Figure 4A**), allows the observation of particle dependent fluorescence (green staining in **Figure 4C**). When the combined fluorescence is overlaid on the contrast image of the lung (**Figure 4D**), one can clearly see that the particle dependent signal is located within the airspace of alveoli. In **Figure 4D**, curved lines having a black color can be identified as capillary vessels, as they form hollow space in the preparation of the cryosection. The rest of the space which has smooth gray color and surrounded by the capillaries is the walls of the alveoli. To confirm that the fluorescence is due to the presence of a dye in the lung cryosection sample, the emission spectra of three different samples was examined. As can be seen from the spectra, in the case of the dye administration within the particles or in free form, there is a fluorescent peak at 754 nm wavelength, which is the character exactly for the Cy7 dye. However, when the dye is delivered via vaterite particles, the intensity of this peak is higher, since the dye is locally concentrated in the particles, and not spread over the entire volume of the tissue. Further evidence of the location of the vaterite particles can be seen when one considers a 3D reconstruction of the confocal images (**Figure 5**). In this image capillaries are clearly visible both in the autofluorescent (red color) and the Cy7 (green color) channels, but for the Cy7 channel there are also regions where granular structures that are clearly absent in the autofluorescence channel. Detectors of the fluorescent signal were tuned in such a way that all fluorescence from the tissue and blood in the picture is red, and all other objects have a green color. Areas where there is no fluorescent signal correspond to the alveolar space, since free space does not give fluorescence when the correct confocal plane is selected to exclude the autofluorescence of the glass slide. Thus, it is clearly seen that the granular structures are concentrated in the alveolar space. Such structures are the colored vaterite particles.

**FIGURE 6:**
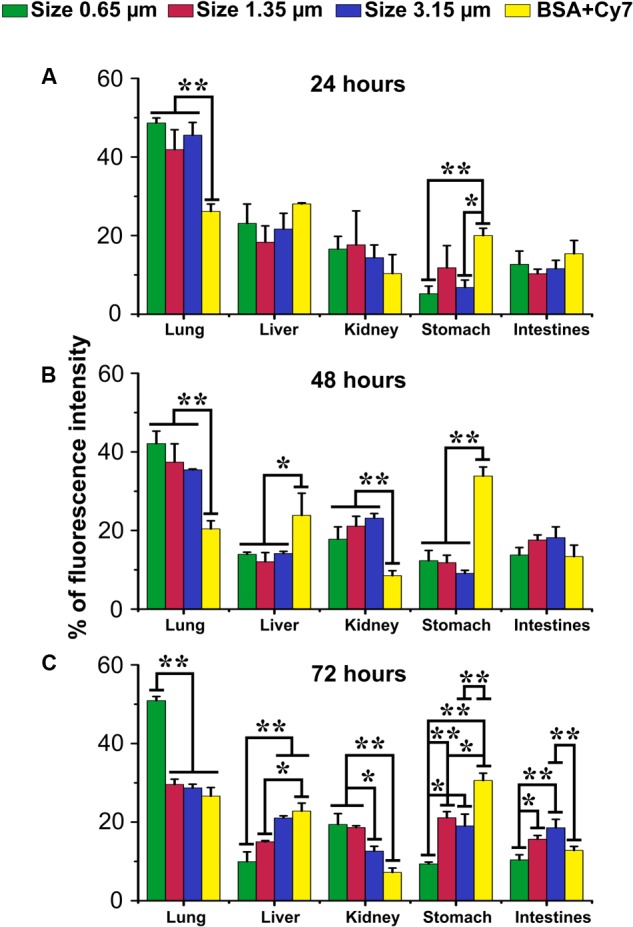
Biodistribution of submicron vaterite particles *in vivo.* Biodistribution within organs of 0.65, 1.35, and 3.15 μm size particles adsorbed with BSA-Cy7 conjugate or BSA-Cy7 alone after **(A)** 24 h, **(B)** 48 h, and **(C)** 72 h intratracheal administration. Data are expressed as mean value ± SD, *n* = 3 mice per group. ^∗^*p* < 0.05, ^∗∗^*p* < 0.01.

To investigate whether particles that enter the lower lung can be retained there and as a result particle dissolution and content release slowed, we examined the delivery of fluorescent compounds to the organs of the mouse. Fluorescence signals from mouse whole body were recorded at 24, 48, and 72 h post intratracheal instillation of 0.65, 1.35, or 3.17 μm vaterite particles loaded with BSA-Cy7 conjugate. Then mice were sacrificed and each of the organs was visualized using the IVIS (**Supplementary Figure S3**) and the resultant fluorescence was quantified (**Figure 6**). The values are given as a percentage of the total fluorescence at the time point so that the relative organ distribution can be determined. As one can see even 72 h after installation half the administered Cy7 is retained within the lungs. Furthermore, tissue distribution through the other organs follows a classical ADME (absorption, distribution, metabolism, and excretion) pathway, first being seen in the liver and then the kidneys. In contrast, free BSA-Cy7 conjugate is moved out of the lung very quickly (even by 24 h) and excreted. Both 1.35 and 3.17 μm are retained in the lung at 24 h, but have almost completely dissipated by 72 h.

**FIGURE 7:**
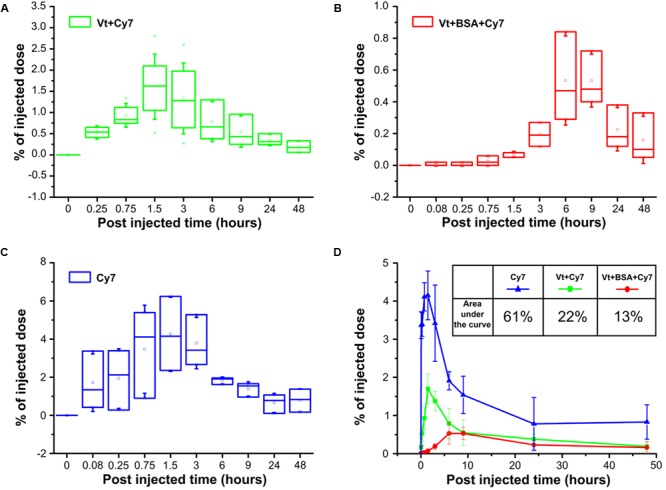
Pharmacokinetics of submicron vaterite particles *in vivo.* Blood samples were analyzed for % of injected dose at indicated time points (5, 15, and 45 min, 1.5, 3, 6, 9, 24, and 48 h) after intratracheally administration of 0.65 μm particles adsorbed with **(A)** Cy7 dye or **(B)** BSA-Cy7 conjugate. **(C)** Fluorescent dye Cy7 alone was used as a control. The dose of fluorescent dye Cy7 was 300 ng for each administration. **(D)** Average percentages of the released fluorescent dye Cy7 to the blood within 48 h post intratracheally administration. The significant differences between intratracheal instillation of free fluorescence dye Cy7 and adsorbed dye or conjugate with BSA into vaterite particles possess a value *p* < 0.01 meanwhile the difference between instillation of vaterite particles with absorbed dye and absorbed conjugate is significant (*p* < 0.05) until 6 h. The inset shows the values of the areas under the curves in the case of three different input substances.

As part of this study, data were obtained examining the pharmacokinetics of delivery both Cy7 dye and BSA-Cy7 conjugate to the blood. Cy7 was instilled into the lungs of mice through a tracheostomy either in its free form or incorporated in the vaterite particles (**Figure 7**). 0.65 μm particles were adsorbed with Cy7 as a model of a low molecular weight bioactive substance (**Figure 7A**), or as a conjugate of BSA and Cy7 as a model of a high-molecular bioactive substance (**Figure 7B**) with free Cy7 as a control (**Figure 7C**). As one can see, in each case there is a maximum concentration of the dye in the blood. For both the single dye adsorbed onto particles and for free Cy7, the maximum concentration is observed 1.5–3 h after administration. Although less total dye is observed in the blood for particle loaded Cy7 over 48 h, presumably as a considerable portion is retained within the lung. For the case of administration of BSA and Cy7 conjugate adsorbed onto the vaterite, there is a time shift for the maximum concentration of the dye in the blood, observed 6–9 h after administration. However, once again the total dye entering the bloodstream in 48 h, as shown by the reduced % under the curve (13% vs. 60%), is much lower (**Figure 7D**). These demonstrate that vaterite particles are capable of providing a slow release mechanism for large biologically active molecules from the lung.

## Discussion

The concept for this study was that by using vaterite particles, because of their biophysical properties, one could generate a porous drug delivery mechanism that could reach the respiratory portion of the lung and provide a prolonged release to the blood. Essential elements of this proposal were that by changing the particle generation technique one could alter the particle size. As from a physics perspective smaller particles should penetrate further into the lung, the use of these techniques should allow us to generate particles that can penetrate all the way to the alveolar surface. This is a critical target for drug delivery, as this is the respiratory portion of the lung, which is a critical component of many lung diseases. In addition, deep penetrance will allow for more effective delivery to the blood. The final element of this proposal was that vaterite particles will interact with the components of the lung lining fluid to become coated, which will reduce dissolution rates and lead to a prolonged release of drug. In the experiments outlined here each of these elements was shown to have some validity.

Vaterite particles are a metastable form of calcium carbonate and tend to transform into a thermodynamically more stable form of calcite (Ogino et al., 1987). Such a transformation is possible due to the simultaneous crystallization and dissolution processes that occur with particles in solution, and can be described in some approximation in terms of a homogeneous theory of nucleation (Gomez-Morales et al., 1996; Parakhonskiy et al., 2014). The rate of these processes vary in different substances. Here, we show that this transformation occurs more readily in saline than in water, which is in agreement with prior studies (Parakhonskiy et al., 2012). However, more importantly for our purposes we observe that incubation of vaterite particles in lung lining fluids, which have a similar salt concentration to saline, considerably slows dissolution (**Figure 2**). A number of studies have shown that protein adsorbs onto the surface of foreign particles that come in contact with biological fluids (Aggarwal et al., 2009; Del Pino et al., 2014; Ke et al., 2017). Vaterite particles are not expected to be an exception in this matter, since they have a negatively charged surface (de Leeuw and Parker, 1998) and have long been known for their ability to adsorb various proteins (Volodkin et al., 2004a). Although a detailed study of this issue has not yet been conducted.

Scanning electron microscopes images in **Figure 2**, devoted to the morphological changes in the vaterite particles within interacting with fractions of the pulmonary surfactant, clearly show the slowing down of the transformation process into calcite. To explain such particles behavior, it should be noted that the large aggregate surfactant fraction is more hydrophobic and contains the small surfactant protein, SP-B and SP-C; while the small aggregate fraction is hydrophilic and contains the large collectin proteins SP-A and SP-D (Griese, 1999). Proteins SP-B and SP-C operate with the principle lipids of the lung lining, phosphatidylcholine, to control surface tension. In solution they exist predominantly as formed micelles, which may limit their ability to interact with other surfaces at a concentration higher than the critical concentration of micelle formation. Due to their hydrophilicity, SP-A and SP-D can interact well with the surface of particles, indeed one of their main functions as collectins is to associate with foreign materials (Wright, 2005). It is reasonable to suppose that these proteins form a corona on the vaterite particle surface that prevents morphological changes, and hence why dissolution was slowest in this fluid. Protein adsorption onto the surface of particles interferes with the dissolution process, which leads to a decrease in the number of ions and, in connection with this, the growth of new crystals slows down. Something similar was shown in (Sergeeva et al., 2015), where the vaterite particles were coated with layers of polyelectrolytes, which resulted in a strong increase in the time for complete recrystallization of the vaterite particles compared to uncoated particles. It was also demonstrated that the time of recrystallization depends on polyelectrolyte layer numbers. Increasing of polyelectrolyte layer number leads to increasing the recrystallization time of vaterite particles (Sergeeva et al., 2015). From the graph in **Figure 2E**, the size of the calcite crystals is seen in the case of incubation of vaterite particles in the large aggregate surfactant fraction to be two times larger than when incubating in the small aggregate surfactant fraction. This appears to indicate that the small aggregate fraction is coating the particles more efficiently and thus slowing down recrystallization to a greater extent. These conclusions are supported by visual examination of the particles themselves by SEM (**Figure 2**) where the formation of a protein corona can be clearly observed. Importantly for our purposes, these studies clearly demonstrate that vaterite particles will have a reduced recrystallization rate when delivered to the lung due to their interaction with the lung lining fluid. This is an important observation if we wish to design a prolonged drug delivery mechanism.

A second goal of our study was to use particle size as a means to increase delivery to the respiratory portion of the lung. It was our proposal that as the opening of the alveolar neck is in the micron to submicron range that particles would need to be smaller than this to enter the very lowest regions of the lung. But it was a concern that such particles might not get that far into the lung as they would be more easily trapped or lost in the upper portions. Therefore, we examined three different particles ranging from 0.65 to 3.15 μm in size (**Figure 1**). All three particles were able to enter the lung and to delivery loaded material, BSA conjugated with Cy7, to the body (**Figure 6**). However, the 0.65 μm particles displayed the most promise as a future delivery mechanism. These particles entered the lung readily (**Figure 3**) and reached the alveolar region (**Figures 4, 5**). Furthermore, they displayed the longest retention time within the lung (**Figure 6**) and favorable pharmacokinetics for a slow release system to the vasculature (**Figure 7**).

One of the most intriguing aspects of this study is the observation that using 0.65 μm vaterite particles one can achieve delivery to the respiratory portion of the lung. A number of lung diseases, including COPD and acute lung injury, have a significant element of inflammation in these areas as a component of their pathophysiology. It is technically difficult to delivery anti-inflammatory drugs to this portion of the lung via the respiratory tract as the bulk of the active component is trapped in the conducting airways, where these compounds may have negative side effects. The results presented here demonstrate that we can achieve significant delivery to the respiratory portion of particles. In concept, this means that drug could be contained in a vehicle that would only release its contents once it had reached the respiratory portion. Furthermore, the reaction with surfactant components will drive a slow release rate of drug which could be useful in the treatment of chronic lung diseases such as emphysema. Further studies need to be performed to determine the efficiency of delivery to the alveoli and the relative distribution that can be achieved. In addition, these were native vaterite particles, and the possibility remains that by coating these particles with polyelectrolytes one may be able to regulate the dissolution rate in the lung lining fluid. In summary, we have provided evidence that using vaterite particles we can generate a novel drug delivery mechanism for the lung that can be used to provide wither pulmonary pharmacology slow release to the systemic circuit.

## Author Contributions

OG, EA-V, GS, DG, and AJG: conception and design, analysis and interpretation, and drafting the manuscript for important intellectual content. OG, OS, SS, SP, NP, and VR: acquisition of data. OG, EA-V, OS, SS, SP, NP, VR, GS, DG, and AJG: final approval of the manuscript.

## Conflict of Interest Statement

The authors declare that the research was conducted in the absence of any commercial or financial relationships that could be construed as a potential conflict of interest.

## Footnotes

1 https://fiji.sc

## Supplementary Material

FIGURE S1 Normalized fluorescence intensity spectrum. The fluorescence peaks of the samples with a maximum at a frequency of 760 nm corresponds to a fluorescent Cy7 dye. The insets show the Cy7 molecular structure.

FIGURE S2 Biodistribution of submicron vaterite particles *in vivo* during 72 h. Figure demonstrates *in vivo* fluorescence images of mouse’s whole body in two views (abdomen and back). Biodistribution after intratracheal instillation of **(A)** 0.65 μm, **(B)** 1.35 μm, **(C)** 3.15 μm size particles with adsorbed BSA-Cy7 conjugate and **(D)** free Cy7 dye.

FIGURE S3 Biodistribution in organs of submicron vaterite particles 72 h post-instillation *ex vivo.* Biodistribution of 0.65, 1.35, and 3.15 μm size particles adsorbed with BSA-Cy7 conjugate or BSA-Cy7 alone after 72 h **(A)** in the lungs, **(B)** in other organs. Data are expressed as mean value ± SD, *n* = 3 mice per group. ^∗^*p* < 0.05, ^∗∗^*p* < 0.01.
