# Supplementary figures and images for: Use of Submicron Vaterite Particles Serves as an Effective Delivery Vehicle to the Respiratory Portion of the Lung
Source: Front Pharmacol. 2018 Jun 4;9:559. doi: 10.3389/fphar.2018.00559 (PMC5994594; doi:10.3389/fphar.2018.00559)

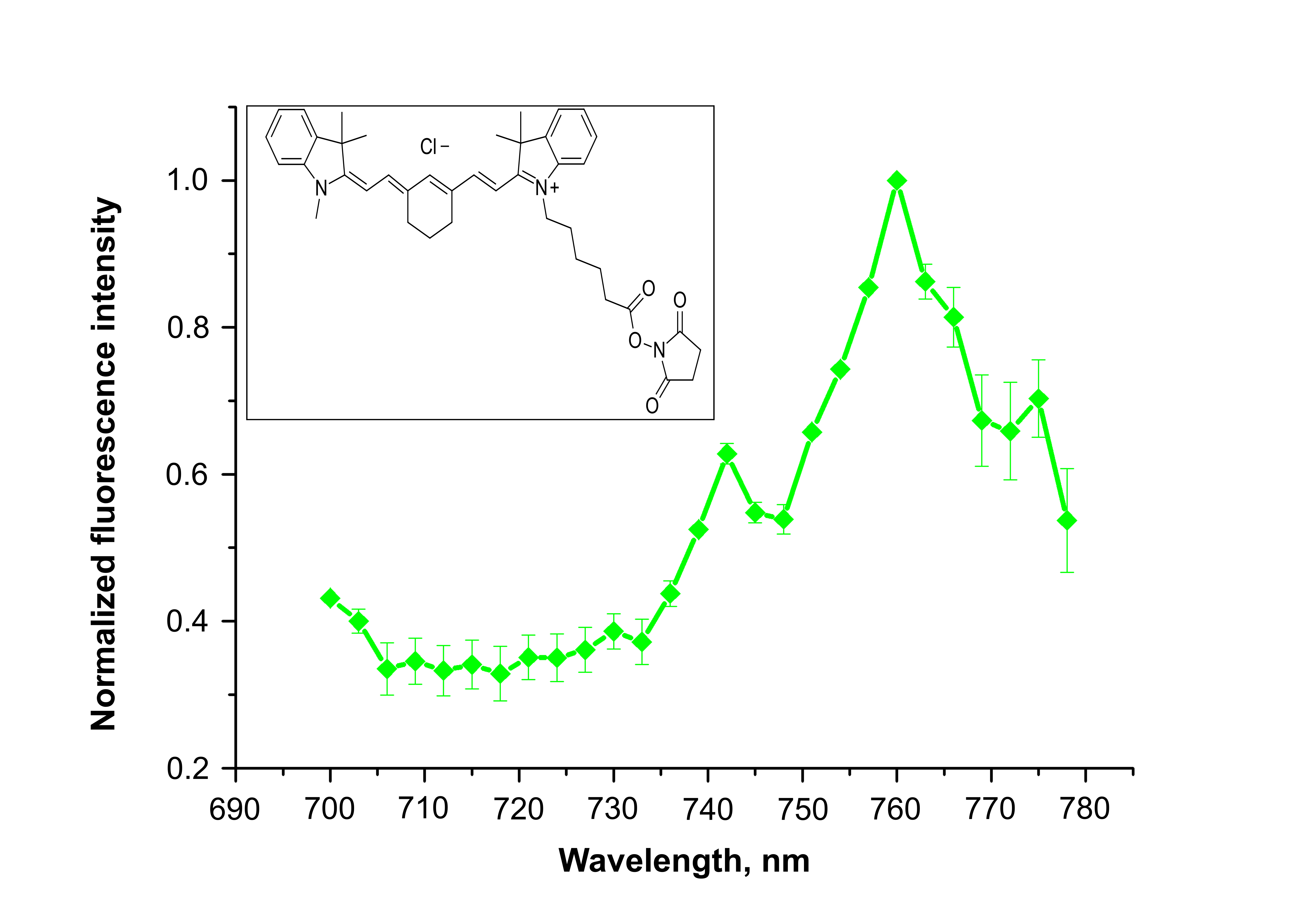

Supplement: FIGURE S1 — Normalized fluorescence intensity spectrum. The fluorescence peaks of the samples with a maximum at a frequency of 760 nm corresponds to a fluorescent Cy7 dye. The insets show the Cy7 molecular structure. [file Image_1.jpg]

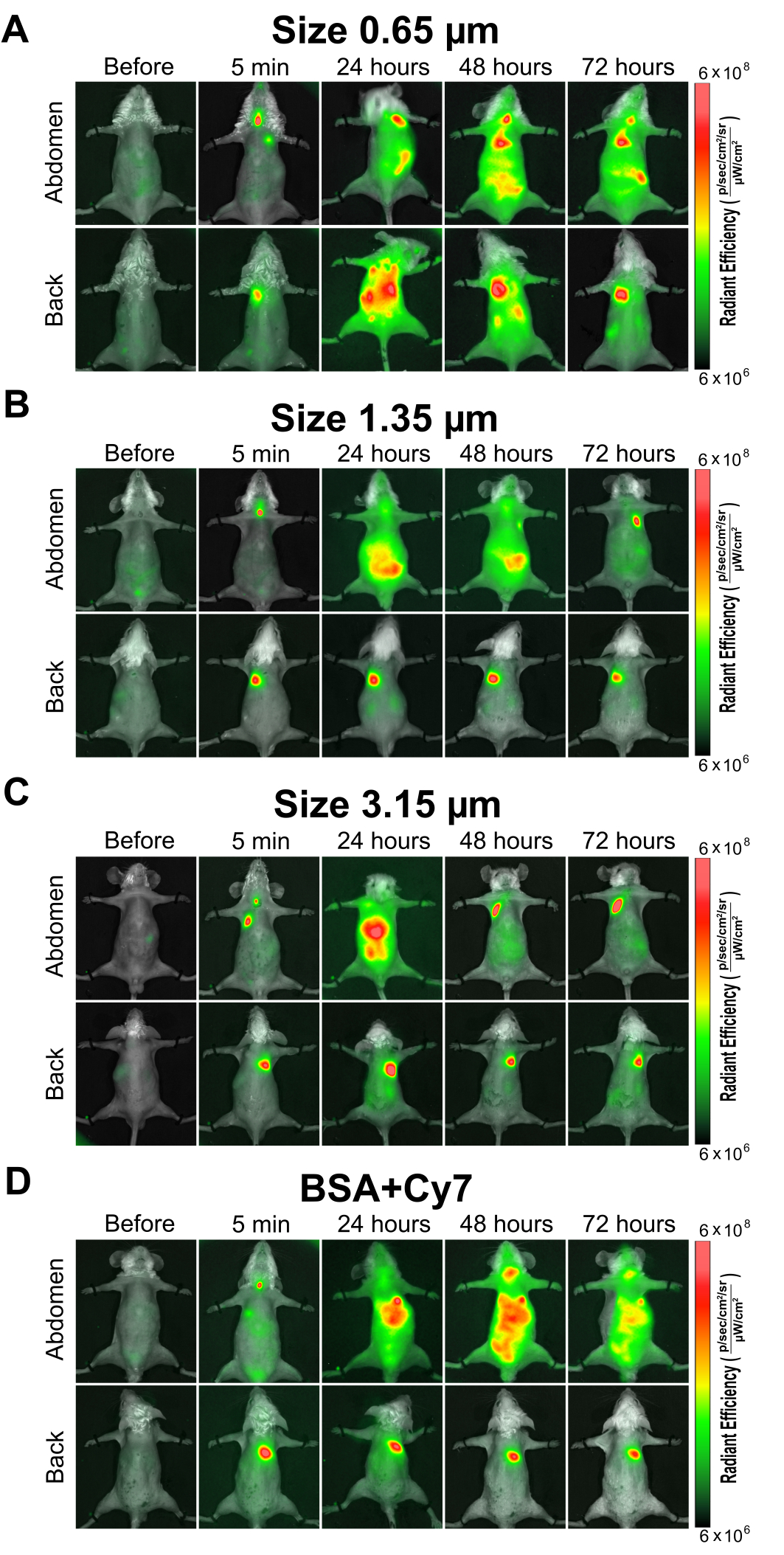

Supplement: FIGURE S2 — Biodistribution of submicron vaterite particles in vivo during 72 h. Figure demonstrates in vivo fluorescence images of mouse’s whole body in two views (abdomen and back). Biodistribution after intratracheal instillation of (A) 0.65 μm, (B) 1.35 μm, (C) 3.15 μm size particles with adsorbed BSA-Cy7 conjugate and (D) free Cy7 dye. [file Image_2.jpg]

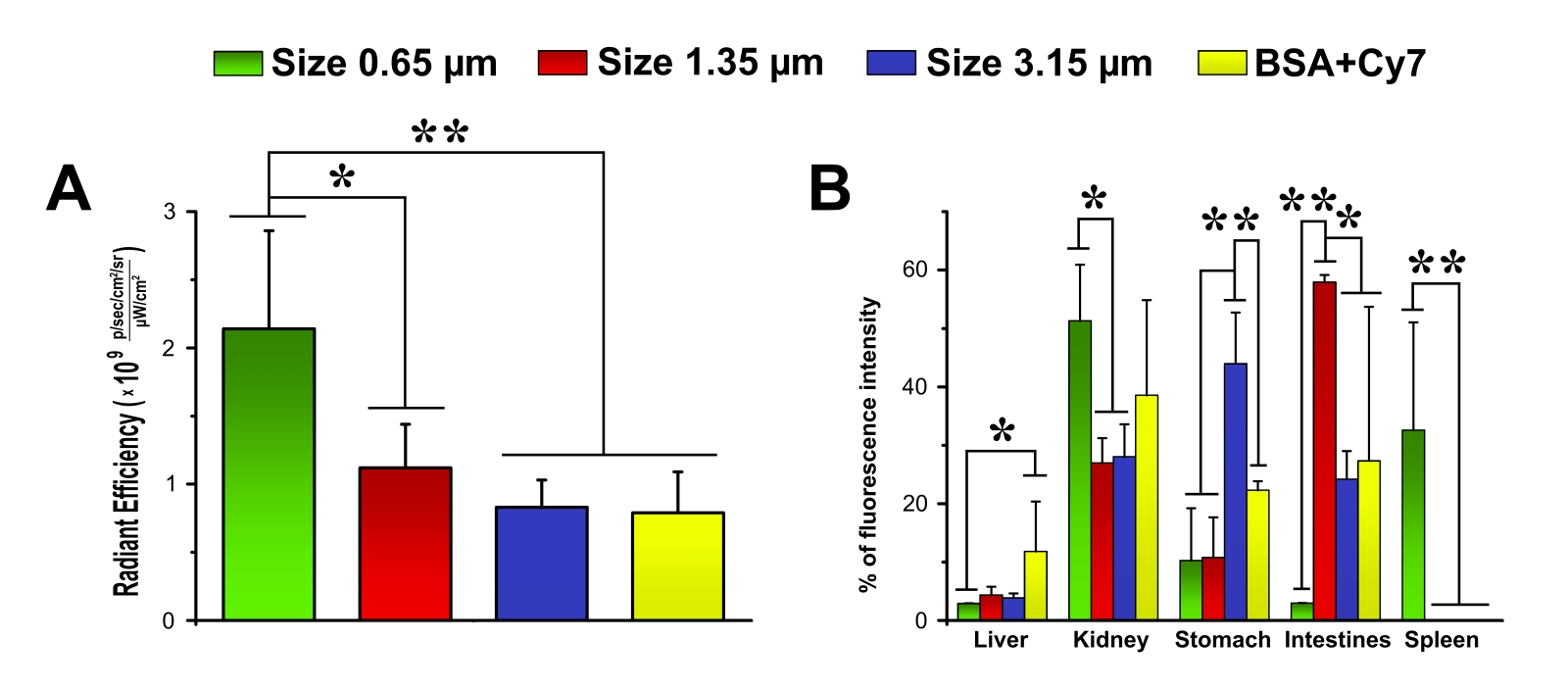

Supplement: FIGURE S3 — Biodistribution in organs of submicron vaterite particles 72 h post-instillation ex vivo. Biodistribution of 0.65, 1.35, and 3.15 μm size particles adsorbed with BSA-Cy7 conjugate or BSA-Cy7 alone after 72 h (A) in the lungs, (B) in other organs. Data are expressed as mean value ± SD, n = 3 mice per group. ∗p < 0.05, ∗∗p < 0.01. [file Image_3.jpg]
